# Two-year follow-up of women with lichen sclerosus treated with laser therapy: A prospective observational study

**DOI:** 10.1007/s10103-026-05062-5

**Published:** 2026-10-13

**Authors:** Volker Viereck, Marianne Gamper, Helena Bischofberger, Irena Zivanovic

**Affiliations:** 1 Blasenzentrum der Frau AG, Frauenfeld, Switzerland; 2 Spital Thurgau, Frauenfeld, Switzerland

**Keywords:** Long-term follow-up, Neodymium:YAG laser, Erbium:YAG laser, Corticosteroid, Sexuality, Lichen sclerosus

## Abstract

This prospective observational study evaluates the 2-year follow-up of a randomized controlled trial comparing a novel dual Nd:YAG/Er:YAG laser therapy with topical corticosteroid therapy for vulvar lichen sclerosus (LS). After the 6-month primary outcome assessment, observation continued with optional further treatment, including a free crossover laser therapy for the initial steroid group. At 24 months, participants were assessed for their subjective symptoms, treatment satisfaction, and maintenance therapy use. Sixty-five of 66 participants underwent four laser sessions during the 24-month follow-up period. The median interval between the final laser treatment and symptom recurrence requiring initiation of maintenance corticosteroid therapy was 7 months (interquartile range [IQR] 2.5–12.5 months). During follow-up, 40 women used maintenance corticosteroid therapy and five decided for an additional top-up laser treatment after a median interval of 18 months (IQR 16–20 months). Overall satisfaction with the treatment regimen was high, and most participants perceived laser therapy as effective. Subjective symptoms remained significantly improved compared with baseline throughout follow-up (p < 0.001). Among sexually active participants, the proportion without dyspareunia increased significantly from 4/31 at baseline to 14/31 at 24 months (p = 0.002). Dual Nd:YAG/Er:YAG laser therapy adjunct to maintenance corticosteroid therapy was well accepted and resulted in sustained symptomatic improvement in women with vulvar LS over 24 months. This combination therapy could represent a potential advance in the long-term management of LS.

## Introduction

Vulvar lichen sclerosus (LS) is a chronic inflammatory disorder of the anogenital skin that requires long-term management because of its persistent course and tendency to relapse. Without adequate treatment, ongoing inflammation may result in progressive architectural changes of the vulva, including scarring, atrophy, fissuring, stenosis, and irreversible distortion of the external genital anatomy. The LS symptoms are dominated by persistent pruritus, burning, soreness, and pain, resulting in substantial impairment of daily activities, sexual function, body image, and overall quality of life [1–5].

Although considerable advances have been made in understanding LS, its pathophysiology remains incompletely understood. Current evidence indicates that disease development is likely driven by a complex interaction between immune-mediated mechanisms, genetic susceptibility, hormonal factors, and environmental influences [4]. As no curative treatment exists, therapeutic strategies aim to achieve sustained suppression of inflammation, relieve symptoms, preserve vulvar architecture, and minimize the risk of long-term complications. Potent topical corticosteroids are considered the standard first-line treatment and have demonstrated substantial efficacy in reducing symptoms and malignant progression [6, 7]. However, a life-long steroid treatment is challenging because of concerns regarding steroid-related adverse effects and reduced adherence to a prolonged topical therapy [4, 8].

Against this background, interest in adjunctive treatment options has increased considerably. Energy-based therapies, particularly laser treatment, have been proposed as a means of improving tissue integrity and supporting long-term symptom control. Fractional carbon dioxide (CO₂), erbium-doped yttrium aluminum garnet (Er:YAG), neodymium-doped yttrium aluminum garnet (Nd:YAG), and diode laser systems have been investigated for their potential to stimulate tissue remodeling through controlled thermal effects, including collagen remodeling, neovascularization, neoangiogenesis, and epithelial regeneration [9–18]. Preliminary studies and randomized trials (RCTs) have reported encouraging clinical outcomes, suggesting that laser therapy may represent a beneficial adjunct to conventional corticosteroid treatment. Nevertheless, currently available evidence remains limited by heterogeneous treatment protocols, small study populations, and insufficient long-term follow-up [2, 15, 19]. Furthermore, experience from laser therapy in stress urinary incontinence suggests that treatment effects may diminish over time, highlighting the importance of evaluating long-term durability [20].

The present study provides the first two-year prospective follow-up of an RCT comparing Nd:YAG/Er:YAG laser therapy with standard first-line topical corticosteroid treatment in women with LS. Following the initial 6-month evaluation, participants were allowed to choose their subsequent treatment strategy, including a free crossover laser therapy for women originally assigned to the steroid arm [9, 21].

The objective of this observational study was to assess the long-term sustainability and safety after laser therapy in women with LS. Further objectives included the evaluation of symptom recurrence, the timing and frequency of topical corticosteroid or top-up laser treatment, and the overall patient satisfaction with both the therapeutic strategy and laser treatment outcomes.

## Materials and methods

### Study design

**Fig. 1:**
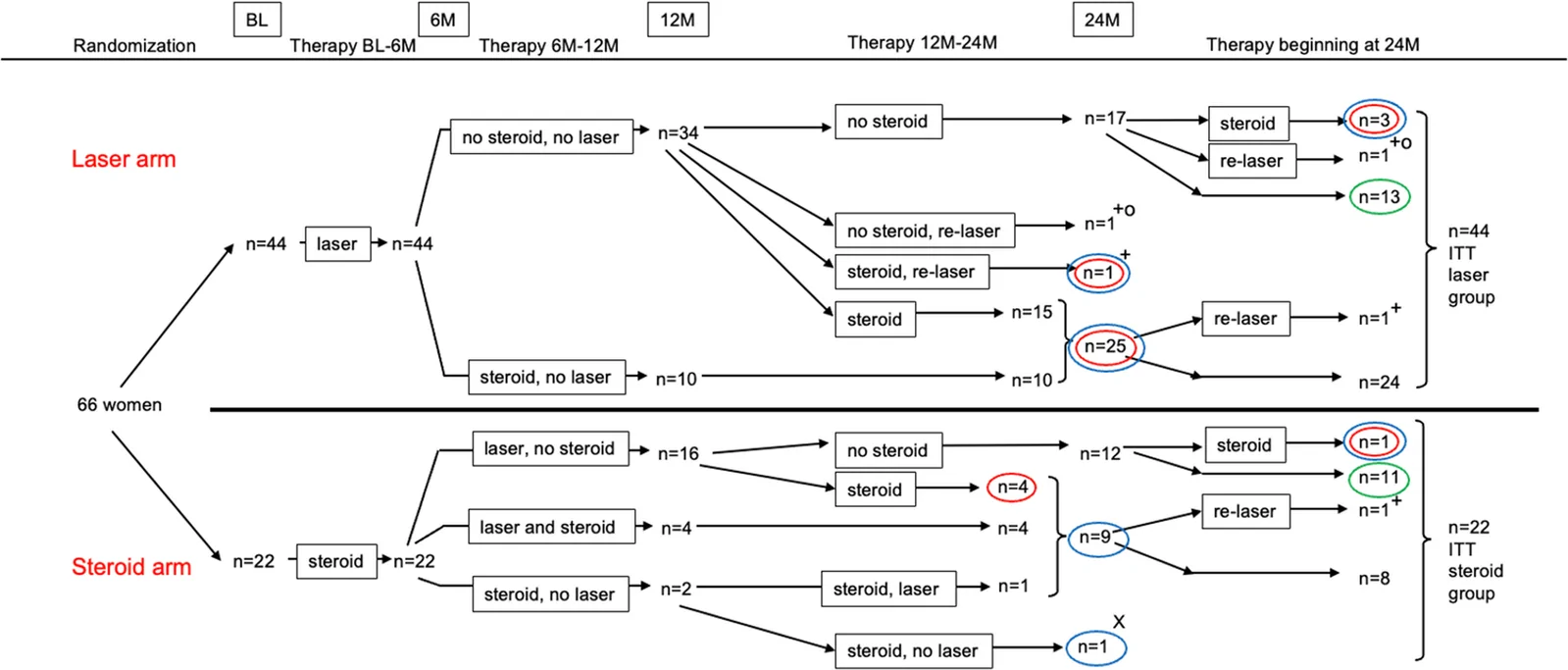
Flow diagram. Randomization at baseline to the laser or steroid arms, outcome evaluation at 6, 12 and 24 months. After 6 months, patients were free to choose their further therapy. Twenty-one women in the steroid arm chose a free crossover laser treatment, 20 between 6 and 12 months, and one between 12 and 24 months. X: 1x no laser therapy; +: 5x top-up laser (= “re-laser”); +o: 2x top-up laser only. Red circled numbers: 34x start with steroid maintenance therapy after laser therapy; blue circled numbers: 40x steroid maintenance therapy; green circled numbers: 24x only laser treatment. BL, baseline; 6 M, 6 months; 12 M, 12 months; 24 M, 24 months; ITT, intention-to-treat

This analysis investigated the two-year prospective observational follow-up data of an investigator initiated, single-center, active-controlled RCT carried out at an ambulatory tertiary referral center. Baseline, 6-month and 12-month data have been published [5, 9, 21]. This study was performed in line with the principles of the Declaration of Helsinki and was registered (www.clinicaltrials.gov, NCT03926299, 24 April 2019). Written informed consent was obtained from all participants included in this study. The study protocol and the CONSORT diagram up to the 6-month follow-up were published [9, 22]. The flow diagram between baseline and 24 months is shown in Fig. 1. At 24 months, subjective outcome was either assessed by a telephone interview or during a clinical visit.

Women with a clinical diagnosis of vulvar LS and a physician-administered clinical LS score ≥ 4 were included [23, 24]. The LS score was validated and had been used in several clinical LS studies [4, 25–28]. Further inclusion and exclusion criteria were published [22]. After randomization, women in the laser arm received four laser treatments with the dual Nd:YAG/Er:YAG laser (FotonaSmooth SP^®^ Spectro laser, Model M021-4AF/3) with the following laser settings: First, non-ablative photothermal Nd:YAG heating was performed with a non-contact R33 handpiece (9 mm spot size) in PIANO mode (5 s pulses) for 5 brushings, with the fluence gradually increased from 70 to 100 J/cm² over subsequent sessions based on patient tolerance [18]. Next, superficial irregularities were ablated with the Er:YAG laser using the R11 handpiece (5 mm spot size) in MSP mode (100 µs pulses) at 2 Hz, with a fluence titrated between 1 and 3 J/cm² according to patient comfort [9, 22]. Local anesthetics were very rarely required but applied if necessary [9]. Women in the steroid arm received de-escalating doses of standardized topical clobetasol 0.05% [9].

After the 6-month visit, the study was continued as a prospective observational study. Treatment options were individually discussed. Patients could choose between no treatment, steroid and/or laser treatment. A concomitant steroid maintenance therapy was always advised. Women in the original steroid arm were offered four free laser treatments (crossover) using the laser settings shown above [9]. All other top-up laser therapies for LS had to be self-paid. Steroid maintenance was defined by a continuous regular local steroid treatment once or twice a week, also in the absence of symptoms [6, 29].

### Outcome measures

The primary outcome of the original RCT was the change of the objective clinical LS score in the laser arm between baseline and 6 months [9]. Secondary outcome measures were the changes of subjective parameters between baseline, 6, 12 and 24 months. At 24 months, subjective symptoms were evaluated by the vulvovaginal symptoms questionnaire (VSQ) [30, 31], the visual analogue scale (VAS) score, the Patient Global Impression of Improvement questionnaire (PGI-I) [32], and three additional questions addressing satisfaction with the laser therapy: (1) subjective feelings after vs. before laser therapy, (2) recommendation of laser therapy, (3) a subjective assessment whether an additional top-up laser treatment would be beneficial or not. VSQ questions 1–16 (symptoms, emotions, life impact) yielded a total VSQ score from 0 to 16 (most severe), VSQ questions 18–21 (sexual impact) a VSQ score from 0 to 4 (most severe). The VAS score described the symptom strength on a scale from 0 to 10 (most severe). Itching, burning and pain yielded a total VAS score from 0 to 30 (most severe), VAS dyspareunia a score from 0 to 10 (most severe). For PGI-I, women were asked to compare their vulvar condition at 6, 12 and 24 months with their condition before the treatment. Treatment satisfaction was rated on a 7-point Likert scale, from − 3 (very much worse) to + 3 (very much better).

### Statistics

The paired Wilcoxon signed-rank test was used to compare matched ordinal measurements obtained from each subject before and after treatment. (Statistics Kingdom, https://www.statskingdom.com/175wilcoxon_signed_ranks.html, accessed February 10, 2026). The original PGI-I numbers without data collapsing were used for statistics. Median and interquartile range (IQR) were calculated with Excel. Paired binary responses before and after therapy were compared using the McNemar’s test (Omni calculator, https://www.omnicalculator.com/statistics/mcnemars-test, accessed February 10, 2026). A p value below 0.05 was considered statistically significant; p values were rounded to 3 digits or indicated as < 0.001 where applicable.

## Results

### Six- and twelve-month data

Baseline, 6- and 12-month data have been published [9, 21]. At 6 months, objective outcomes for the clinical LS score, atrophy and subjective treatment satisfaction were significantly better in the laser arm than in the steroid arm, but all other improvements were similar in both arms. After 6 months, the majority of participants of the original steroid arm wanted a free crossover laser treatment. At 12 months, the therapeutic effect persisted in the laser arm, and the crossover laser therapy improved LS score, atrophy and patient satisfaction leading to a similar sustained improvement in both study arms (ITT analysis).

### Two-year follow-up

The subjective assessment at 24 months showed that 65 of the 66 enrolled patients have received a laser therapy consisting of four laser sessions. One patient originally allocated to the steroid arm was satisfied with steroid maintenance therapy and therefore did not want a laser treatment (Fig. 1, symbol X). All 44 patients in the primary laser arm received laser therapy between baseline and 6 months. Twenty-one of 22 patients originally in the steroid arm opted for a crossover laser therapy after 6 months (Fig. 1).

During the 2-year follow-up period, five women underwent a self-paid top-up laser treatment: two patients between 12 and 24 months and three patients at the 24-month visit (Fig. 1, symbol +). The time interval between the last laser treatment and the top-up laser treatment was 18 months (median, IQR 16–20; range 13–23 months). The reasons for repeat laser treatment were itching (2x), dyspareunia (2x), and pain (1x).

### Steroid maintenance therapy

Sixty of 65 women stopped steroid application during laser therapy, all 44 women in the laser arm and 16 women in the steroid arm (Fig. 1). Only five patients chose to continue steroid maintenance therapy in parallel with crossover laser treatment.

After laser therapy, 34 women started with steroid maintenance therapy (Fig. 1, red circled numbers), 24 wanted no steroid treatment (Fig. 1, green circled numbers), and 2 had a top-up laser only (Fig. 1, symbol + o). The time interval between the last laser treatment and the initiation of steroid maintenance was 7 months (median, IQR 2.5–12.5; range 0–21 months). The main symptom triggers for re-starting steroid therapy were itching (15x), atrophy (6x), fissures (2x), urinary tract infection (2x), incontinence (1x), pain (1x), redness (1x), stenosis (1x), and medical recommendation (5x). A total of 61% (40/66) patients used steroid maintenance therapy at the 24-month follow-up (Fig. 1, blue circled numbers).

### Patient satisfaction

Overall satisfaction with laser therapy was very high. All 65 patients who received laser therapy (100%) answered affirmative to the question “Do you feel better today than before laser therapy?”. Sixty-four of 65 patients (98%) recommended laser therapy to others. Sixty of 65 patients (92%) believed that a top-up laser therapy would be beneficial; two were negative and three left the question unanswered.

PGI-I at 24 months indicated that 53/66 (80%) patients felt “very much better” or “much better” regarding their LS condition compared to 38/66 (58%) at 6 months or 49/66 (74%) at 12 months. Patients demonstrated a statistically significant improvement on the PGI-I scale from 6 to 12 months (*p* = 0.019) which was characterized by a widening upper interquartile range (Table 1). This improvement was due to increased satisfaction among the 21 women who underwent crossover laser therapy [21]. Thereafter, scores stabilized from 12 to 24 months (*p* = 0.252). Overall, the comparison between the 6- and 24-month follow-ups confirmed a robust, statistically significant global improvement (*p* < 0.001; Table 1).

**Table 1:** PGI-I, VAS and VSQ at baseline, 6, 12 and 24 months

Outcome measure	Paired (*n*)	BL	6 M	12 M	24 M	*p* value ^a^	Effect size ^a^ (*r* [95% CI])
**PGI-I**	66	NA	2 (1–2)	2 (1.25–2.75)	2 (2–3)	**< 0.001**	0.42 [0.20, 0.60]
**VAS total** (itching, burning, pain)	66	10 (6–14)	4 (2–6)	2 (1–5)	2 (0–3)	**< 0.001**	0.82 [0.77, 0.86]
**VSQ total** (Q1-16)	66	7 (6-10.75)	4 (2–8)	2.5 (2-5.75)	2 (1-3.75)	**< 0.001**	0.81 [0.73, 0.86]

^a^p value and effect size: Wilcoxon signed rank test (paired) compare the initial and the 24-month time points; r, non-parametric effect size using the total sample (*n* = 66); data for scores are presented as median (IQR); PGI-I, Patient Global Impression of Improvement; VAS, visual analogue scale; VSQ, vulvovaginal symptoms questionnaire; Q1-16, specific questions of the VSQ; BL, baseline; 6 M, 6 months; 12 M, 12 months; 24 M, 24 months; NA, not applicable. Statistically significant values are shown in bold

### Subjective symptom scores (VAS and VSQ) and adverse events

Subjective symptoms assessed by the VAS and the VSQ scores significantly improved over the entire observation period from baseline to 24 months (Table 1). Up to 24 months, no laser-related adverse events or malignant progression were reported throughout the entire observation period.

### Sexual activity and dyspareunia

During the 2-year follow-up, 31/66 women (47%) reported at all visits of being sexually active with a partner, 24/66 (36%) reported no sexual activity at any visit, and 11/66 (17%) reported sexual activity at some visits only.

Analysis of the sexually active sub-cohort (*n* = 31) demonstrated highly significant long-term improvements across both continuous severity distributions and categorical complete symptom resolution. VAS dyspareunia and VSQ sexual impact significantly improved from baseline to 24 months (Table 2a). The proportion of women without dyspareunia (VAS = 0) increased significantly from 13% at baseline to 45% at 24 months (Table 2b). Similarly, the proportion of women without subjective sexual problems (VSQ Q18-21 = 0) increased from 9.7% at baseline to 48% at 24 months (Table 2b).

**Table 2:** Sexual outcome

a							
Outcome measure	Paired (*n*)	BL	6 M	12 M	24 M	*p* value ^a^	Effect size ^a^ (*r* [95% CI])
**VAS dyspareunia**	31	5 (2.5-7)	3 (0–5)	2 (0-4.5)	1 (0-3.5)	**< 0.001**	0.77 [0.56, 0.89]
**VSQ sexual impact** (Q18-21)	31	3 (2–3)	3 (1–3)	2 (1–3)	1 (0–3)	**0.002**	0.55 [0.29, 0.75]

b						
Outcome measure	Paired (*n*)	BL	6 M	12 M	24 M	*p* value ^b^
**VAS: no dyspareunia**	31	4/31 (13%)	11/31 (35%)	11/31 (35%)	14/31 (45%)	**0.002**
**VSQ: no sexual impact**	31	3/31 (9.7%)	7/31 (23%)	7/31 (23%)	15/31 (48%)	**< 0.001**

Data are shown for the 31 sexually active women. (a) ^a^p value and effect size: Wilcoxon signed rank test (paired) compare the baseline and the 24-month time points; r, non-parametric effect size using the total sample (*n* = 31); data for scores are presented as median (IQR). (b) ^b^p values: McNemar’s test (paired) compare the baseline and the 24-month data; data for scores are expressed as count/total (%). VAS, visual analogue scale; VSQ, vulvovaginal symptoms questionnaire; Q18-21, specific questions of the VSQ; VAS: no dyspareunia, VAS dyspareunia = 0; VSQ: no sexual impact, VSQ sexual impact (Q18-21) = 0. BL, baseline; 6 M, 6 months; 12 M, 12 months; 24 M, 24 months. Statistically significant values are shown in bold

Over the 2-year observation period, the proportion of sexually active women decreased slightly: 40/66 (61%) at baseline, 36/66 (55%) at 6 months, 36/66 (55%) at 12 months, and 35/66 (53%) at 24 months. LS was the reason for abstaining from partnered sexual activity in 25% of cases overall.

## Discussion

The present study provides, to our knowledge, the first extended follow-up evaluation of dual Nd:YAG/Er:YAG laser therapy in women with vulvar LS over a 24-month period. Except for one patient, all participants (65/66) underwent laser treatment. All 44 women initially randomized to the laser arm received four laser treatments between baseline and 6 months while 21 women initially randomized to the steroid arm applied a controlled topical corticosteroid therapy during the first 6 months followed by four laser treatments (crossover) starting at 8 months. By 12 months, no differences were observed between the original study arms (intention-to-treat analysis) [21].

Laser therapy was associated with an improvement of LS [9]. However, these benefits appeared to diminish over time. Among the 60 women without concomitant corticosteroid therapy during the laser treatment phase, 34 started steroid maintenance therapy after a median interval of seven months. In comparison, five women with distressing symptoms requested a self-paid follow-up laser treatment after a median of 18 months. At the 24-month follow-up, 61% of the participants were using corticosteroid therapy. This ratio increased from 12 months, when only 24% of the women used steroid maintenance therapy (Fig. 1). During the 2-year observation period, 36% of the patients – who were symptom-free – wanted to extend the beneficial effect of the laser therapy and therefore refrained from steroid maintenance therapy, contrary to our recommendations.

Maintenance application of topical corticosteroids was in accordance with the current clinical guidelines for long-term management of LS, and – together with regular follow-ups – was recommended to avoid malignant progression [6, 7]. The concept of adjunct laser treatment followed by standard steroid maintenance treatment has the advantage of prolonged symptom relief, an extended steroid-free interval, and a reduced risk of malignant progression due to maintenance therapy. Therefore, this combined approach may represent a novel therapeutic concept for the long-term management of LS. In this study, the combined treatment regime resulted in a sustained subjective improvement when compared with baseline. Additionally, sexually active women reported improved sexual function.

Study limitations must be acknowledged. The observational duration of two years introduces potential confounding factors that may influence patient-reported outcomes, including changes in health priorities and the emergence of other conditions. Indeed, our clinical observation suggested that over time, patients may shift their focus away from LS to other urogenital or systemic concerns, potentially altering their perception of LS-related symptoms. In general, confounding factors are inherent limitations affecting all outcomes of long-time observational studies. The assessment of exclusively subjective long-term outcomes might constitute an additional limitation. However, since LS is predominantly defined by severe and distressing symptoms, the subjective outcome reported by the patient represents a clinically relevant measure of the long-term effectiveness of the treatment. Further inherent limitations are the relatively small sample size, no randomized design after the 6-month follow-up, no objective outcome at the 24-month follow-up such as histology, clinical LS score or physical assessment, and different timings of laser treatments between the original laser group / the crossover laser group and the top-up laser treatments.

A strength of this study is the complete follow-up of all participants over a 2-year observational period. Despite the recurrence of symptoms in a substantial proportion of patients, subjective outcomes indicated high patient satisfaction at 24 months. The results demonstrated observational within-participant trends rather than the independent effect of the laser treatment. Nevertheless, laser therapy appeared to be crucial for the initial alleviation of the highly distressing LS symptoms. The achieved effects could subsequently be sustained by the patients’ choice of further adjunct treatments (laser and/or steroids). Patients generally perceived the chosen combined therapeutic approach favorably, and a high proportion of 92% were convinced of the additional benefits of undergoing a top-up laser treatment. Post-study medical history showed that at least nine more patients received a top-up laser treatment after the 24-month follow-up period, resulting in at least 21% (14/66) women willing to pay for a further laser treatment. Importantly, with the individually combined laser/steroid treatment approach no cases of premalignancies were identified during the observation period.

## Conclusion

Laser therapy for vulvar LS was generally well accepted by patients and provided symptomatic relief; however, recurrence of symptoms over time was common, and steroid maintenance was resumed after a median of seven months, or a top-up laser therapy was resumed after a median of 18 months. Professional societies and clinical guidelines recommend established topical corticosteroid therapy as the standard of care treatment, with the primary goals of preventing disease progression and reducing the risk of malignant transformation. Current evidence supports the use of dual Nd:YAG/Er:YAG laser therapy as part of a multimodal adjunct management strategy, also including maintenance therapy with topical corticosteroids and regular clinical follow-ups to reduce the risk of progression.

## Data Availability

The data that support the findings of this study are available from the corresponding author upon reasonable request.
